# Separators for Li-Ion and Li-Metal Battery Including Ionic Liquid Based Electrolytes Based on the TFSI^−^ and FSI^−^ Anions

**DOI:** 10.3390/ijms150814868

**Published:** 2014-08-22

**Authors:** Marija Kirchhöfer, Jan von Zamory, Elie Paillard, Stefano Passerini

**Affiliations:** 1 Institute of Physical Chemistry, University of Muenster, Corrensstraβe 28-30, Muenster 48149, Germany; E-Mails: marija.kirchhoefer@uni-muenster.de (M.K.); jan.vonzamory@uni-muenster.de (J.Z.); 2 Helmholtz Institute Ulm, Electrochemical Energy Storage, Albert-Einstein-Allee 11, 89081 Ulm, Germany and Karlsruhe Institute of Technology PO Box 3640, Karlsruhe 76021, Germany

**Keywords:** ionic liquids, lithium metal, separator, Li-ion

## Abstract

The characterization of separators for Li-ion or Li-metal batteries incorporating hydrophobic ionic liquid electrolytes is reported herein. Ionic liquids made of *N*-butyl-*N*-methylpyrrolidinium (PYR_14_^+^) or *N*-methoxyethyl-*N*-methylpyrrolidinium (PYR_12O1_^+^), paired with bis(trifluoromethanesulfonyl)imide (TFSI^−^) or bis(fluorosulfonyl)imide (FSI^−^) anions, were tested in combination with separators having different chemistries and morphologies in terms of wetting behavior, Gurley and McMullin number, as well as Li/(Separator + Electrolyte) interfacial properties. It is shown that non-functionalized microporous polyolefin separators are poorly wetted by FSI^−^-based electrolytes (contrary to TFSI^−^-based electrolytes), while the ceramic coated separator Separion^®^ allows good wetting with all electrolytes. Furthermore, by comparing the lithium solid electrolyte interphase (SEI) resistance evolution at open circuit and during cycling, depending on separator morphologies and chemistries, it is possible to propose a scale for SEI forming properties in the order: PYR_12O1_FSI > PYR_14_FSI > PYR_14_TFSI > PYR_12O1_TFSI. Finally, the impact the separator morphology is evidenced by the SEI resistance evolution and by comparing Li electrodes cycled using separators with two different morphologies.

## 1. Introduction

Even though the use of renewable resources, such as wind, solar and tidal energy has grown in the last years and is foreseen to allow providing most of the energy consumption by 2030 [1,2,3], most of our energy consumption still derives from fossil fuels. In fact, the decrease of this proportion would require an increase of stationary energy storage capabilities, as most renewable sources provide electricity in an intermittent way, incompatible with the electric network needs, as well as a switch to electric transportation. For both purposes, larger battery packs, as compared to portable electronics, are needed, raising larger safety concerns.

Indeed, if secondary Li-ion batteries offer among the highest energy densities, with values up to 800 Wh·L^−1^ for the latest generation of 18,650 cells, they are based on a system which is not thermodynamically stable. Indeed, lithiated graphite, generally used as anode, is unstable toward most chemicals and its operation is made possible by the kinetic passivation of its surface by electrolyte degradation products, forming a protective layer commonly called “Solid Electrolyte Interphase” (SEI) [4] by analogy with Li metal [5]. At the same time, layered oxide cathodes typically used in Li-ion cells, such as LiCoO_2_, LiNi_0.33_Mn_0.33_Co_0.33_O_2_ or LiNiO_2_, are known to release oxygen in case of overcharge or overheating [6,7].

In addition, the electrolytes are made of a mixture of cyclic alkyl carbonates, usually ethylene carbonate (EC), and volatile linear alkyl carbonates with low flash points such as dimethyl carbonate (T_F(DMC)_ = 17 °C) or diethyl carbonate (T_F(DEC)_ = 25 °C) and the use of the low thermal stability [8,9] salt LiPF_6_ brings the additional concern of generating HF in case of contact with the atmosphere or water traces above 60 °C [10,11]. For all these reasons, the operation of Li-ion batteries above 50 °C is problematic, especially as high temperatures can induce the failure of the SEI this resulting in the direct exothermic reaction of lithiated graphite with the electrolyte [12], possibly chain reactions, releasing rapidly the stored energy in what is generally referred to as “thermal runaway”.

On the other hand, ionic liquids (ILs) have attracted a growing attention as electrolytes for Li-based batteries, due to their non-flammability, negligible vapor pressure, high thermal and electrochemical stabilities [13,14,15,16,17]. Indeed, if the use of graphite requires, for most IL-based electrolytes, the addition of additives, such as VC, for forming the SEI [18,19], some ILs based on the bis(fluorosulfonyl)imide (FSI^−^) anion possess intrinsic SEI forming properties [20,21,22,23], as well as higher conductivities [20,24,25,26] as compared to the more commonly used bis(trifluoromethanesulfonyl)imide (TFSI^−^) based ILs. These electrolytes, in addition to allowing higher operation temperatures for Li-ion batteries, and thus easier heat management for large packs, also allow good reversibility of the Li metal electrode both for TFSI^−^ [27] and FSI^−^-based electrolytes [26,28], which enables their use in emerging Li-metal battery technologies, such as Li–metal oxide, Li–air, and Li–S [17].

Besides the electrolyte, the separator plays an important safety role in lithium batteries by preventing the physical contact between the electrodes, while allowing the Li^+^ ions transport through its pores, filled with the electrolyte. Tri-layered polyolefin separators including a polyethylene (PE) layer sandwiched between two polypropylene (PP) layers are commonly used in Li-ion batteries due to their chemical inertia and to the safety feature they offer, the so-called “shut-down effect” as, in case of overheating, the PE layer melts, loosing its porosity (*i.e*., mechanically blocking the Li^+^ ion movement), while the PP layer prevents large dimensional changes until its own melting, thus preventing short-circuits [29,30,31,32]. Nevertheless, these separators still suffer from rather limited temperature range, with an onset for shrinkage around 100 °C [32]. Thus, in recent years, separators with “zero shrinkage”, made of polymer coated with inorganic particles have been introduced, such as Separion^®^, allowing higher safety [33]. As Li-ion electrolytes are hydrophilic, these separators also allow for a better wetting as compared with polyolefin separators, which makes them especially suitable for large battery cells. Other materials, chemically less inert, such as cellulose have also been investigated [34,35] as cheaper alternatives to dry-stretched or wet processed commercial Li-ion separators.

However, in most reports on ionic liquids for Li-ion or Li-metal batteries, glass fiber (GF) separators are used, while their thickness of *ca.* 300 µm, *versus* *ca.* 25 µm for commercial separators is not adapted for commercial applications. If the ionic liquid family is wide, the most promising ILs comprise the TFSI^−^ and FSI^−^ anions, which confer them hydrophobic properties, which *a priori*, makes them more compatible with polyolefin separators as compared with hydrophilic conventional electrolytes, even though their higher viscosity, raises questions. Nevertheless, no study can be found concerning the use of separators for IL-based electrolytes in Li-ion or Li-metal batteries.

If for Li-ion batteries the contact between the porous electrodes and the separator is limited to a small fraction of the tridimensional composite electrode total surface, which limits the interfacial reactivity, a different situation occurs in Li-metal batteries as the separator is in contact with a larger fraction of the total electrode surface, making the study of the interfacial reactivity more crucial. Indeed, the pre-existing “native” SEI, originating from the passivation of Li in dry air, might crack at the contact with the separator, as it is the case for semi-crystalline solid polymer electrolytes [36], which induces increased reactivity and electrolyte consumption for SEI “self-repair”. Moreover, the main drawback of the Li electrode is the deposition of Li under the form of dendrites, which prevented so far the successful commercialization of liquid electrolyte-based, Li-metal secondary batteries, while commercial Li-metal polymer batteries do not include any separator. The formation of dendrites is not only related to the total current density [37,38], but, also, presumably, to the local variation of current densities linked to inhomogeneities within the SEI [39], which could also originate from the presence of a separator. Thus, separator’s morphology and chemical composition are both likely to influence the properties of the Li/(electrolyte + separator) interface.

In this work, we investigated combinations of four different hydrophobic IL-based electrolytes with eight separators in terms of wettability, conductivity and behavior *versus* Li metal. Two commercial polyolefin microporous separators (Celgard^®^), as well as a ceramic-coated membrane (Separion^®^), were used. In addition, polyamide and cellulose-based separators were tested in order to investigate the effect of the chemistry on the ensemble (separator + electrolyte). For comparison, high porosity separators (non woven mats) including two glass fiber separators, as well as a PP separator, were also investigated.

## 2. Results and Discussion 

### 2.1. Separator Characterization

**Figure 1:**
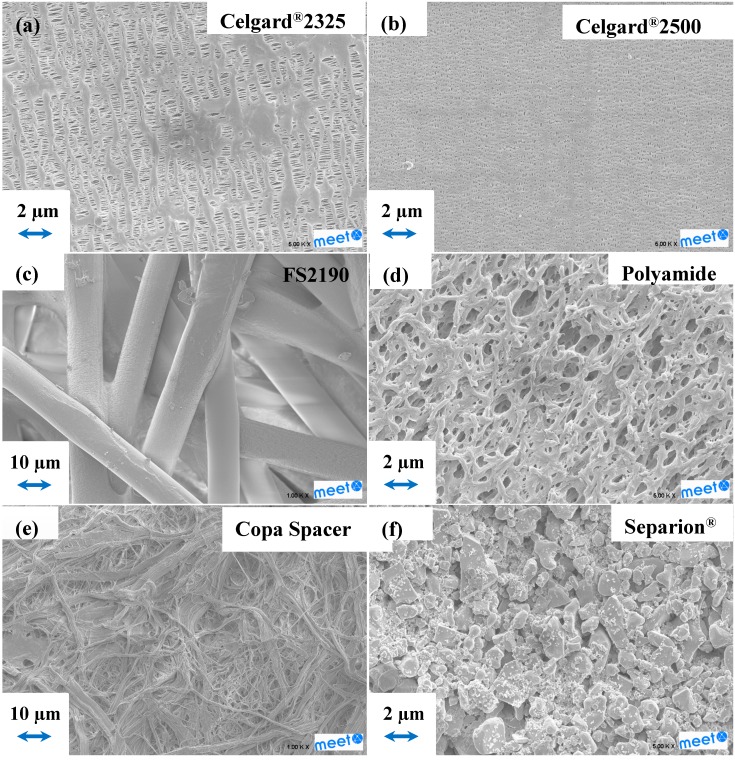
Scanning electron microscopy (SEM) images of (**a**) Celgard^®^2325 (PP/PE/PP); (**b**) Celgard^®^2500 (PP); (**c**) Freudenberg FS2190 (PP); (**d**) Polyamide; (**e**) Copa spacer (cellulose); and (**f**) Separion^®^ (PET/ceramic).

Figure 1 shows the SEM images of the separators, which basic properties are reported in Table 1. The Celgard^®^ microporous polyolefin separators (Figure 1a,b) exhibit typical dry-streched morphologies, where the pore size of the monolayer separator Celgard^®^2500 is larger than that of the outer layer of Celgard^®^2325. The scanning electron microscopy (SEM) image of FS2190, shown in Figure 1c, reveals large fibers of *ca.* 10 µm diameter. In Figure 1d, it can be seen that the polyamide membrane has a tridimensional porous structure, probably originating from a wet processing. The cellulose “Copa Spacer” (Figure 1e) is constituted of non-woven fibers. Finally, only the inorganic outer layer of Separion^®^ can be seen in Figure 1f.

The Gurley numbers, (*i.e.*, the time for 100 cm^3^ of air to flow through 1.0 square inch under a pressure difference of 12.96 mbar) of the various separators are listed in Table 1. The microporous polyolefin Celgard separators show lower air permeability than the other separators, which values are coherent with those from the supplier (Celgard^®^2500: 200 s; Celgard^®^2325: 620 s). The measurement was not possible for Freudenberg FS2190 due to its high permeability. Despite their thickness, Whatman^®^GF/F exhibits a Gurley number of 2.3 s and Whatman^®^GF/C of only 1.0 s. The non-woven cellulose separator also shows a low permeability, while the ceramic coated separator Separion^®^ and the polyamide membrane exhibit intermediate values.

**Table 1:** Composition, thickness and air permeability (Gurley numbers) of the separators.

Separator	Composition	Thickness (µm)	Gurley Number (s)
Celgard^®^2325 (Polypore)	PP/PE/PP	27	570.0
Celgard^®^2500 (Polypore)	PP	27	180.1
FS2190 (Freudenberg and Co.KG)	PP	176	–
Polyamide 0.2 µm (Sartotius Stedium Biotech GmbH)	Polyamide	116	31.9
Copa Spacer (Spez. Papierfabrik Oberschmitt GmbH)	Cellulose	50	6.5
Separion® (Evonik)	Ceramic on PET	28	22.8
GF/C (Whatman)	Glass fiber	283	1.0
GF/F (Whatman)	Glass fiber	359	2.3

### 2.2. Wetting of the Separators

**Figure 2:**
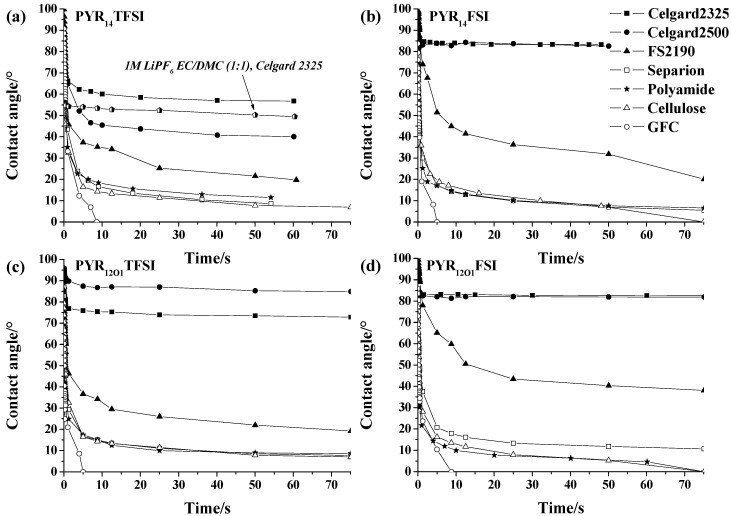
Evolution of the contact angle of drops of 10 mol % LiTFSI electrolytes deposited on the separators: (**a**) PYR_14_TFSI (1M LiPF_6_ in EC:DMC (1:1) + Celgard^®^2325 included for comparison); (**b**) PY_14_FSI; (**c**) PYR_12O1_TFSI; and (**d**) PYR_12O1_FSI.

The wettability of a membrane depends on several factors, such as porosity and surface roughness and, for a given electrolyte, on its chemical affinity for the membrane material, as well as its viscosity. The evolutions of the contact angle of electrolyte drops deposited onto the separators are shown in Figure 2a–c, with the ensemble (1M LiPF_6_, EC:DMC (1:1) + Celgard^®^2325) reported in Figure 2a for comparison. For all electrolytes, the GF/C separator shows the fastest wetting (similarly to GF/F, not shown) as a result of its open porosity. FS2190, which possesses even higher air permeability, does not allow for as fast a wetting, showing that, even if the electrolytes are hydrophobic, they have a better affinity for the hydrophilic SiO_2_ surface than for that of polyolefin. This is especially obvious for the Celgard^®^ separators, which exhibit the slowest wetting performance, as a combined effect of poor surface affinity and low porosity. A major difference between TFSI^−^ and FSI^−^ is evidenced with these latter separators. If, for PYR_14_TFSI and PYR_12O1_TFSI, the contact angle slowly decreases with time (Figure 2a,c), similarly to the conventional and low viscosity electrolyte 1M LiPF_6_ in EC:DMC (1:1), this is not the case for PYR_14_FSI and PYR_12O1_FSI (Figure 2b,d), for which the contact angle remains remarkably constant. Despite their lower viscosities, FSI^−^-based electrolytes show a poorer affinity with polyolefin separators, which could be linked to the lack of hydrophobic perfluorinated carbons in these ILs, despite their overall hydrophobicity. Although PYR_14_TFSI and PYR_12O1_TFSI-based electrolytes are both able to slowly wet the Celgard^®^ separators (Figures 2a,b), PYR_14_TFSI exhibits lower contact angles and a much faster wetting, which can be related only to the lack of ether function in the side chains since its viscosity is higher [40]. The behavior toward the more hydrophilic separators are more difficult to interpret, however, a general scale of hydrophobicity, based on the wettability results of the three polyolefin separators can be proposed: FSI^−^ < TFSI^−^ and PYR_1(2O1)_^+^ < PYR_14_^+^.

The more hydrophilic Separion^®^ and the polyamide membrane showed in most cases similar trends and a good affinity with all electrolytes. The hydrophilic cellulose-based Copa Spacer showed a rapid decrease of contact angles and a complete absorption of PYR_14_^+^-based electrolytes within 80 s, while PYR_12O1_^+^ electrolytes absorption was slightly slower.

Overall, the hydrophobicity of the ILs does not favor polyolefin separators in terms of wetting, although they can be wetted by TFSI-based electrolytes. Among the thin commercial separators tested, Separion^®^, thanks to its hydrophilicity, allows for the fastest wetting.

### 2.3. Influence of the Separator on the Conductivity

The influence of the separator on the conductivity of the ensemble (electrolyte + separator) is reflected through its MacMullin number *N*m, the ratio of the resistivity of the combination (electrolyte + separator) alone by the resistivity of the electrolyte. It can be seen in Table 2 that the commercial separators including a safety feature (shut-down for Celgard^®^2325 and “zero shrinkage” for Separion^®^) exhibit the highest *N*m, although in line with Li-ion requirements [33]. For Celgard^®^2325, only PYR_14_TFSI:LiTFSI was able to wet the separator properly in the conditions of this study, which led to a *N*m similar to that of conventional Li-ion (*ca.* 10) [33] while the values obtained with PYR_12O1_TFSI probably result from a poor wetting as no vacuum was applied during the tests. Interestingly, Celgard^®^2500 combines well with PYR_14_TFSI-based electrolytes, with a rather low *N*m of 5, which makes it a good alternative to GF separators because its lower thickness makes it less detrimental to battery energy density and internal resistance. For PYR_14_FSI-based electrolyte, Separion^®^ is the best compromise between resistivity and thickness. Among the non-woven separators, Freudenberg FS2190 exhibits a *N*m around 2–4 while the lowest *N*m were measured for the GF separators, although it has to be pointed out that, if SiO_2_ has been reported to enhance conductivity in aprotic electrolytes [41,42,43] it could as well be an experimental artifact linked to the compressibility of these separators during the measurement of their *N*m.

**Table 2:** Calculated McMullin numbers (*N*m) for 0.1 mol % LiTFSI electrolytes.

Separator	PYR_14_TFSI	PYR_1(2O1)_TFSI	PYR_14_FSI	PYR_1(2O1)_FSI
Celgard^®^2325	11	27 *	/	/
Celgard^®^2500	5	36 *	/	/
Freudenberg FS2190	3	4	2	3
Polyamide	6	3	2	3
Copa Spacer	3	4	3	4
Separion^®^	9	10	7	11
Whatman^®^GF/C	1	1	1	1
Whatman^®^GF/F	1	1	1	1

* Probably resulting from incomplete wetting of the separator.

### 2.4. Static Evolution of the Solid Electrolyte Interphase (SEI) Resistance

**Figure 3:**
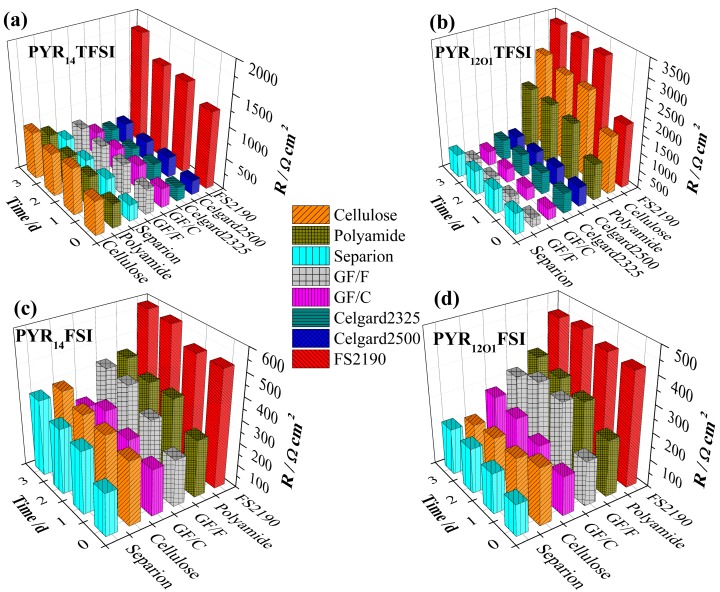
Evolution of the SEI resistance of Li electrodes in contact with 10% mol LiTFSI electrolytes combined with different separators (**a**) PYR_14_TFSI; (**b**) PY_12O1_TFSI; (**c**) PYR_14_FSI; and (**d**) PYR_12O1_FSI.

The evolutions of the SEI resistances, at 21 °C, in contact with the different combinations (electrolyte + separator), are shown in Figure 3. The FSI^−^-based ILs led to much lower resistances as compared with TFSI^−^-based ILs. Among the separators, FS2190 led to the highest resistances, especially when compared to the other polyolefin separators, which can be attributed to the large diameter of its constituting fibers, more likely to induce important damage to the native SEI and subsequent repair [36] by contact. If it was not possible to measure SEI resistance in combination with FSI^−^ based electrolytes, due to their poor wetting, Celgard^®^ membranes, overall, led to the lowest resistances for PYR_14_TFSI, probably due to their relatively high chemical inertia, combined with their low surface roughness as compared to FS2190. This latter allows comparing the electrolytes in combination with a low reactivity separator inducing *a priori* significant SEI cracking, which suggests that the effectiveness of the electrolytes for SEI building/repairing follows the order: PYR_12O1_FSI > PYR_14_FSI > PYR_14_TFSI > PYR_12O1_TFSI. This confirms the good SEI forming properties of FSI^−^ while the higher reactivity of PYR_12O1_^+^ [40] seems to be effective only when combined with FSI^−^, PYR_12O1_TFSI exhibiting the worst behavior toward SEI building. However, the latter material shows comparable SEI resistance to those of PYR_14_TFSI in the case of the GF, Celgard^®^ and Separion^®^ separators.

The cellulose separator led to comparatively high SEI resistances for TFSI-based electrolytes, whereas with PYR_12O1_FSI and PYR_14_FSI the resulting resistances were among the lowest. It should be pointed out that the morphology of the separator has a greater influence than its chemistry as, if cellulose and polyamide lead to larger SEI resistances as compared to that of Celgard^®^ separators, for the TFSI-based electrolytes (Figure 3a,b), their reactivity is compensated by the SEI building properties of the FSI-based electrolytes. The resulting SEI resistances (Figure 3c,d) are in the same range than those obtained with GF separators. In contrast, with the worst SEI forming electrolyte, PYR_12O1_TFSI, these are among the highest. In particular, for all electrolytes, the SEI resistances are lower than those measured for the more inert, but rougher, FS2190. The results obtained with these two reactive separators, however, confirm the relative efficiencies of the electrolytes concerning their SEI forming properties deduced from the polyolefin separators results. Separion^®^ led to relatively low resistances in all cases, with, remarkably, among the lowest measured resistances for FSI-based ILs.

### 2.5. Lithium Plating/Stripping Tests

The symmetrical cells used for the SEI evolution study were then subjected to galvanostatic cycling at 0.1 mA·cm^−2^, reversing the polarity every hour, for 100 cycles at 40 °C. These cells were periodically subjected to impedance measurements (at 21 °C) to follow the evolution of the electrolyte resistance and the SEI resistance.

#### 2.5.1. Glass Fiber Separators

**Figure 4:**
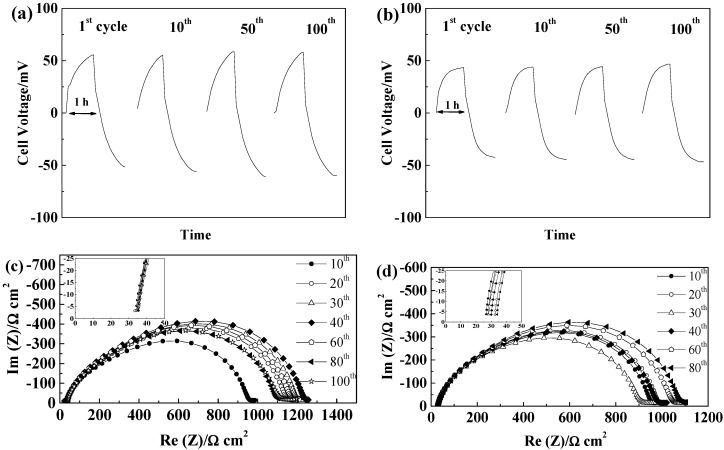
Evolution of PYR_14_TFSI-based electrolyte cells during cycling at 40 °C and 0.1 mA·cm^−2^ (**a**) Voltage profiles of a GF/F cell; (**b**) Voltage profile of a GF/C cell; (**c**) Evolution of the impedance spectra at 21 °C of a GF/F cell; and (**d**) Evolution of the impedance spectra at 21 °C of a GF/C cell.

GF separators allow extended cycling of lithium with both PYR_14_TFSI [44] and PYR_14_FSI^−^ based electrolytes [29,31]. For these separators, the voltage profiles, illustrated in Figure 4a,b, follow those typically linked to the extension of concentration gradients with time and neither steady-state potentials nor Sand time [45] are reached within one hour. If, for the thicker GF/F separator, the cell polarization is higher than for GF/C, we observe only minimal voltage evolution with cycling, showing that, for 100 cycles, neither SEI nor electrolyte are significantly modified. As seen in Figure 4c, the SEI resistance first increases and then slightly decreases. Rather similar values of SEI resistances are observed for the two GF separators, with variations from cell to cell and slightly lower values obtained, in general, with GF/C separators. The electrolyte resistance, corresponding to the high frequency intercept with the real axis showed in the insert of Figure 4c,d, increases slightly over cycling, but always being small as compared to the SEI resistance (at 21 °C). Overall, the mass transport is more limiting than the resistance of the electrolyte and the influence of the SEI variations on the voltage profiles is minimal within the duration of the test. The effect of the separator thickness can be seen in the evolution of electrolyte resistances, with the thicker GF/F leading to almost no change while GF/C leads to limited electrolyte resistance increase, linked to the lower amount of electrolyte contained.

**Figure 5:**
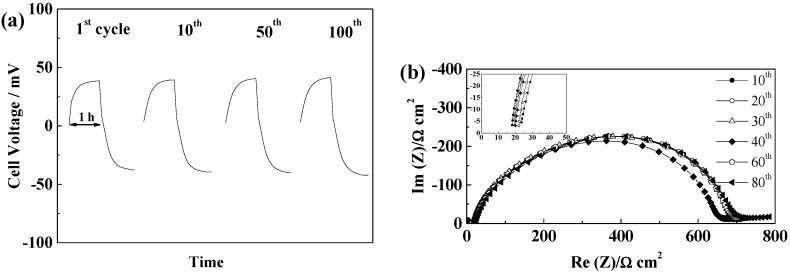
Evolution during cycling at 40 °C and 0.1 mA·cm^−2^ of a PYR_12O1_TFSI-based cell incorporating a GF/C separator (**a**) Voltage profile of a GF/C cell; (**b**) Evolution of the impedance spectra at 21 °C of the GF/C cell shown in (**a**).

The PYR_12O1_TFSI-based electrolyte led to rather similar behavior, with stable end of cycle voltages around 40 mV for PYR_12O1_TFSI/GF/C, as a result of a very stable and low resistance SEI, as seen in Figure 5a,b.

**Figure 6:**
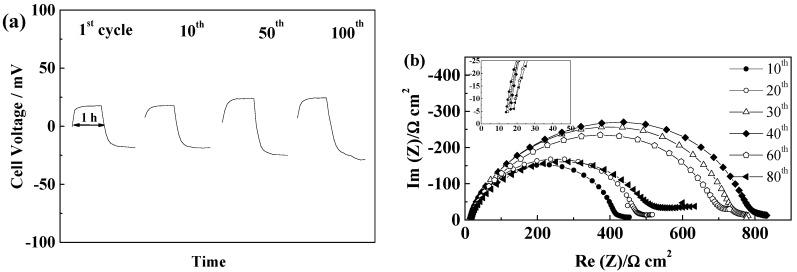
Evolution during cycling at 40 °C and 0.1 mA·cm^−2^ of a PYR_12O1_FSI-based cell incorporating a GF/C separator (**a**) Voltage profile of a GF/C cell; and (**b**) Evolution of the impedance spectra at 21 °C of the GF/C cell.

The FSI^−^-based electrolytes led to lower cell voltage as a result of the lower mass transport resistance of the electrolytes, resulting in low steady-state voltages reached rapidly, as illustrated in Figure 6 for PYR_12O1_FSI, with a slight increase followed by stabilization and cell voltages below 25 mV. The resistance of the electrolyte (see insert in Figure 6b) increases slightly over cycling, while the SEI resistance first increases prior to decreasing slightly above its starting value. The PYR_14_FSI based electrolyte led to slightly higher cell voltage (not shown) around 0.3–0.4 V both with GF/C and GF/F separators.

#### 2.5.2. Celgard^®^2500 and Celgard^®^2325

**Figure 7:**
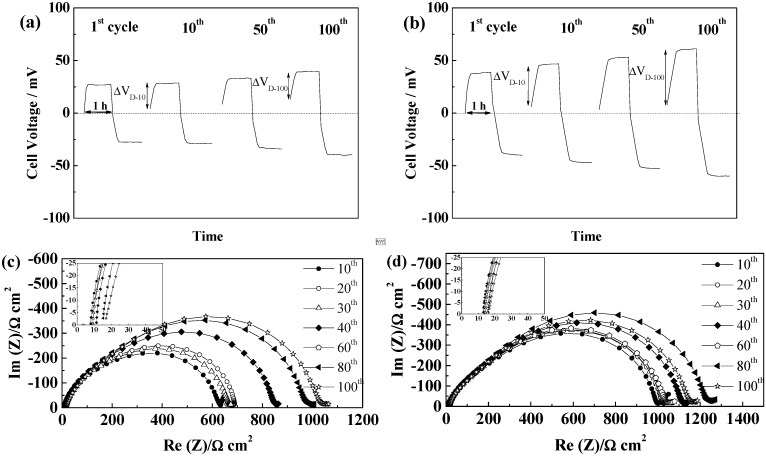
Evolution of PYR_14_TFSI-based electrolyte cells during cycling at 40 °C and 0.1 mA·cm^−2^ (**a**) Voltage profiles of a Celgard^®^2500 cell; (**b**) Voltage profile of a Celgard^®^2325 cell; (**c**) Evolution of the impedance spectra at 21 °C of a Celgard^®^2500 cell; and (**d**) Evolution of the impedance spectra at 21 °C of a Celgard^®^2325 cell.

For the microporous separators Celgard^®^2500 and Celgard^®^2325, only the TFSI^−^ based ILs led to proper wetting and subsequent cycling of the cells. As seen in Figure 7a,b, the two separators led to similar voltage profiles with PYR_14_TFSI, with steady-states reached quickly, in accordance with their low thickness. The cycling voltages were lower as compared with GF separators, especially for Celgard^®^2500. The lower thickness of these separators allows reaching the steady state faster (and lower values), an increase of SEI resistance resulting in this case to a significant increase of ohmic drop, seen as voltage drop when the current is reversed. However, the voltage increase after that first step, linked to Li^+^ mass transport by diffusion (noted ΔV_D_), is not overly modified by the SEI changes.

In the case of Celgard^®^2325, we observe in Figure 7b a slow increase of cell voltage from 55 to 77 mV over 100 cycles, while the SEI resistance (Figure 7d) was rather constant over the whole 100 cycles. In this case, it seems that the lower porosity of the separator induces more marked mass transport limitations, as ΔV_D-100_ > ΔV_D-10_, which in this case, increases during cycling whereas the electrolyte resistance is almost constant.

**Figure 8:**
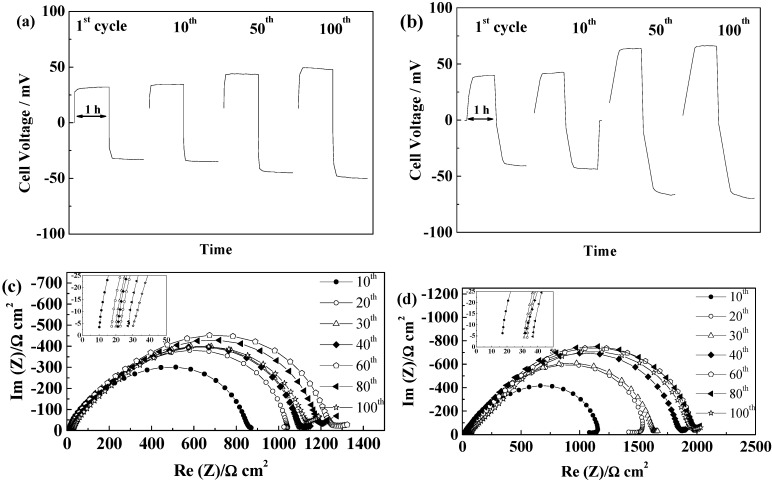
Evolution of PYR_12O1_TFSI-based electrolyte cells during cycling at 40 °C and 0.1 mA·cm^−2^ (**a**) Voltage profiles of a Celgard^®^2500 cell; (**b**) Voltage profile of a Celgard^®^2325 cell; (**c**) Evolution of the impedance spectra at 21 °C of a Celgard^®^2500 cell; and (**d**) Evolution of the impedance spectra at 21 °C of a Celgard^®^2325 cell.

For PYR_12O1_TFSI, the vacuum applied during pouch cell sealing allowed good electrolyte wetting of the Celgard^®^ separators and, as a result, the cell voltages were lower as compared with PYR_14_TFSI, in accordance with the higher conductivity of the electrolyte (See Table 3), as illustrated in Figure 8. With this electrolyte, although the variation of the voltage profiles resembles that of PYR_14_TFSI, with a more marked increase of cell voltage with Celgard^®^2500. However, the resistances of the electrolyte (Figure 8c,d), initially rather low, increase to higher values than those obtained either with PYR_14_TFSI, or using 10 times thicker GF separators. Nevertheless, the cells reached 100 cycles and finally led to similar plating voltage as for GF separators. This confirms the poorer SEI forming properties of PYR_12O1_TFSI, as compared to PYR_14_TFSI, which is especially visible with thin separators as a result of the smaller electrolyte reservoir they constitute.

**Figure 9:**
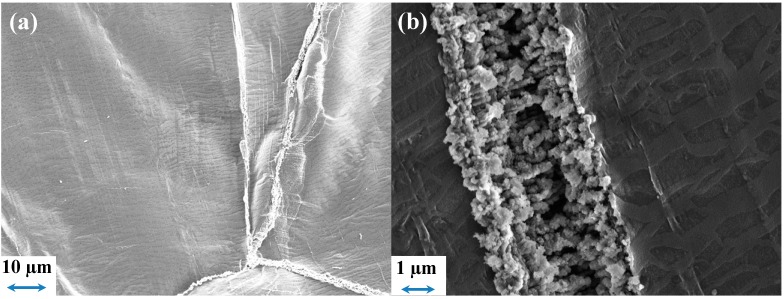
SEM images of the Li surface after 100 cycles, using a 10 mol% LiTFSI PYR_14_TFSI and a Celgard^®^2325 separator.

For PYR_14_TFSI combined with Celgard^®^2325, the SEM images of the Li surface after cycling are shown in Figure 9a,b, indicate that, if the Li surface is not overly modified as a whole, some cracks of *ca.* 1–2 µm can be seen, within which inhomogeneous Li deposition seems to occur. The rest of the Li surface still presents a marble-like texture, which is “native” on the Li foil before cell assembly. Li deposition occurs through this unmodified part of the SEI, finally inducing some cracks of limited impact (although a limited SEI resistance decrease is seen in Figure 7d), despite the possible shortcuts for Li^+^ transport through the generated SEI cracks. In this case, there is no obvious link between the separator morphology and the crack’s pattern, in accordance with the smooth morphology of the separator while the overall volumes variation over cycling could be at the origin of these cracks.

#### 2.5.3. Freudenberg FS2190

**Figure 10:**
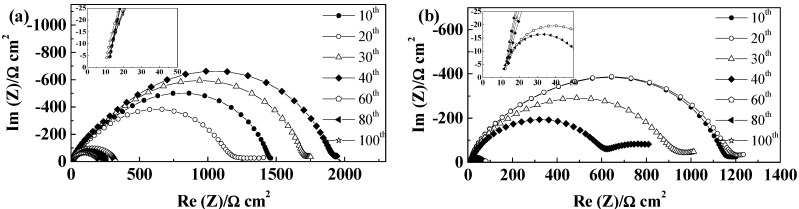
Evolution of the impedance spectra at 21 °C of Freudenberg FS2190 cells during cycling at 40 °C and 0.1 mA·cm^−2^ (**a**) PYR_14_FSI cell; and (**b**) PYR_12O1_FSI cell.

Overall, Freudenberg FS2190 led to short-circuits for many cells, especially those with FSI^−^-based electrolytes, which could be linked to the poor affinity of PP with the electrolyte. Indeed, as seen with the evolution of the SEI resistance, the large FS2190 fibers induce extended cracking of the “native” SEI, which could *a priori* be repaired, given the good SEI forming properties of the FSI^−^ based electrolytes. However, the poor wetting of the fibers by FSI- based ILs might induce poorer access and increased inhomogeneities in local current densities in the vicinity of the fibers in contact with Li and punctual shorts-circuits during cycling. The presence of dendrites was confirmed by the EIS measurements, showing first a growing impedance, more marked in the case of PYR_14_FSI than for PYR_12O1_FSI, followed, in many cells, by sudden decreases, due to dendrites growth, as illustrated in Figure 10 (although no short is visible in the impedance spectra).

**Figure 11:**
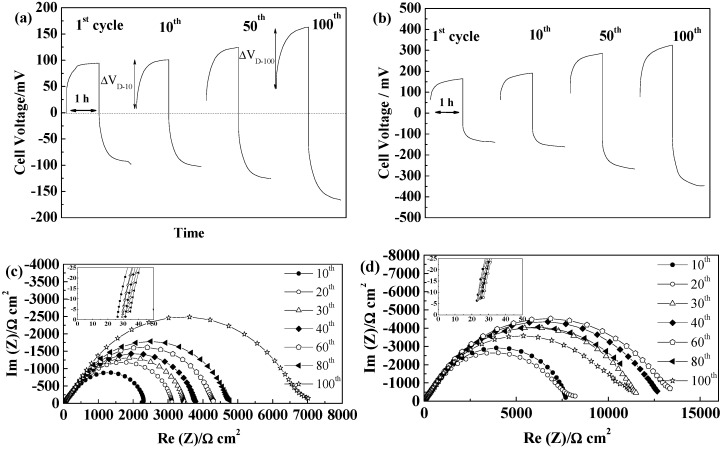
Evolution of Freudenberg FS2190 cells during cycling at 40 °C and 0.1 mA·cm^−2^ (**a**) Voltage profiles of PYR_14_TFSI cell; (**b**) Voltage profile of a PYR_12O1_TFSI cell; (**c**) Evolution of the impedance spectra of the PYR_14_TFSI cell; and (**d**) Evolution of the impedance spectra at 21 °C of the PYR_12O1_TFSI cell.

For TFSI-based electrolytes, cycling without short circuits could be obtained. In combination with PYR_14_TFSI (Figure 11a), the cells showed a continuous increase of cell voltage, starting from 100 mV to reach 167 mV after 100 cycles, as a result of the higher initial SEI resistance and its dramatic increase upon cycling shown in Figure 11c. In this case also, an increase of ΔV_D_ can be observed with cycling in addition to increased ohmic drop.

The use of PYR_12O1_TFSI showed even higher cell voltages, from 147 to 307 mV resulting from an even more dramatic SEI resistance growth (Figure 11d).

**Figure 12:**
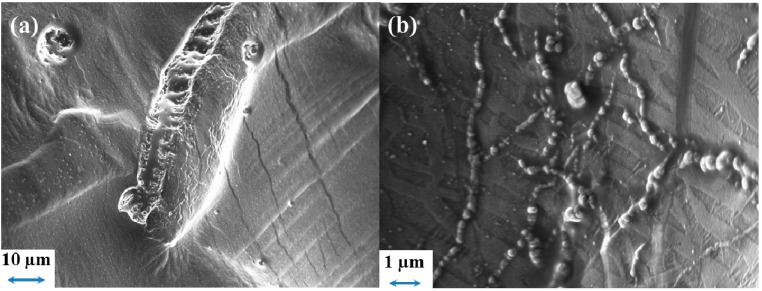
SEM images of the Li surface after 100 cycles, using a 10 mol% LiTFSI PYR_14_TFSI and a Freudenberg FS2190 separator.

To confirm the impact of the separator morphology on the SEI/electrolyte interface, some SEM images of the Li foil from the PYR_14_TFSI cell were acquired and are shown in Figure 12. Some large features with similar dimensions to that of the PP fiber of FS2190 can be seen in Figure 12a. From the images it is observed a change of surface morphology around these features probably due to the accumulation of electrolyte degradation product, which had previously been suggested to lead to additional mass transport limitation during longer term cycling [44]. In other areas, less damaged, smaller features (alignment of small globular dendrites) can also be seen (Figure 12b), however, the surface is still mostly unchanged and the marble-like original morphology mostly preserved. One can suppose that the thickening of the SEI around the damaged area would have induced an increase in current density, triggering dendrite growth as a result of Li^+^ local depletion.

#### 2.5.4. Separion^®^

**Figure 13:**
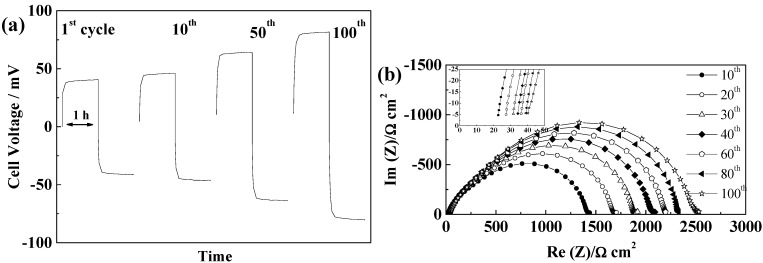
Evolution of a (PYR_14_TFSI + Separion^®^) cell during cycling at 40 °C and 0.1 mA·cm^−2^ (**a**) Voltage profiles; and (**b**) Evolution of the impedance spectra at 21 °C.

The inorganic composite separator Separion^®^ allows Li cycling in combination with PYR_14_TFSI-based electrolyte with, however, the steady increase in cell voltage from 41 to 74 mV over 100 cycles shown in Figure 13a. This is probably due to the continuous increase of the SEI resistance, concomitant with an increase of the electrolyte resistance, as seen in Figure 13b. The inorganic particles at the surface of Separion^®^, although smaller than the fibers of FS2190 are likely to induce well dispersed cracks within the SEI, which could explain the continuous increase of the SEI and electrolyte resistance, as a result of a marked reactivity when using TFSI-based ILs.

**Figure 14:**
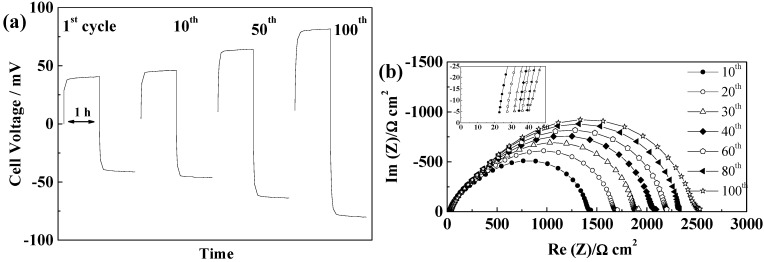
Evolution of Separion^®^ cells during cycling at 40 °C and 0.1 mA·cm^−2^ (**a**) Voltage profiles of a PYR_14_FSI cell; (**b**) Voltage profile of a PYR_12O1_FSI cell; (**c**) Evolution of the impedance spectra at 20 °C of a PYR_14_FSI cell; and (**d**) Evolution of the impedance spectra at 20 °C of a PYR_12O1_FSI cell.

In the case of the PYR_12O1_TFSI, high initial cell voltages were obtained and after few cycles, depending on cells, unstable voltage profile, resulting from pronounced interfacial reactivity. Short circuits could be seen in most cells, which illustrate further the lower SEI forming ability of this electrolyte, although we cannot exclude that stable cycling could have been reached with different “native” SEI or improved interface stabilization. If Separion^®^ allows cycling with PYR_14_TFSI, a continuous increase of SEI resistance is seen for PYR_12O1_TFSI, thus resulting in poor cycling performance. Thus, it can be summarized that the rough and non-flexible surface of Separion^®^ significantly impact the SEI evolution during cycling. On the other hand, the electrolytes with better SEI building ability, PYR_14_FSI and PYR_12O1_FSI, allowed rather stable performance with Separion^®^, as illustrated in Figure 14. With PYR_14_FSI, an increase followed by stabilization of the SEI resistance is observed while almost no changes in electrolyte conductivity are observed (Figure 14c), which results in the stable cell voltage during cycling seen in Figure 14a. It can be noticed that only the ohmic drop increases in this case, but not the diffusive part.

PYR_12O1_FSI presents excellent cycling behavior, as seen in Figure 14b, when combined with Separion^®^, as a combination of its high conductivity, good SEI formation ability, good affinity for the separator, and low thickness of the ensemble. Steady-state current were reached within minutes and the steady-state voltage did not vary in 100 cycles, showing that, even if the SEI resistance increases over cycling (at 20 °C) (Figure 14d). At the cycling temperature, no influence of these change can be seen in the voltage profiles while the total resistance of the two SEI stays below 800 Ω cm^2^ with a slight increase followed by a limited decrease. It is worth pointing out that the combination Separion + PYR_12O1_FSI allowed outstanding performance in these conditions, which show that, even when the “native” SEI is strongly damaged, and especially in this case, with stable plating voltages in the 10–15 mV range, and confirms its excellent SEI forming ability.

#### 2.5.5. Cellulose “Copa Spacer”

**Figure 15:**
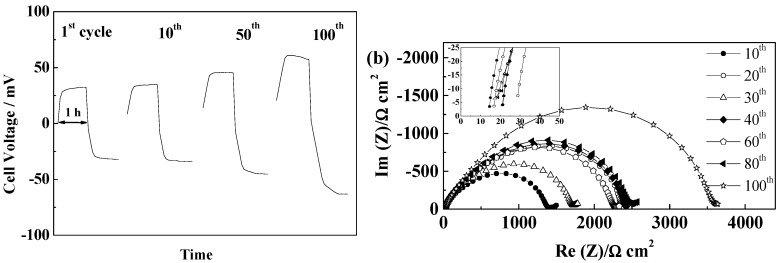
Evolution of a (Cellulose + PYR_14_TFSI) cell during cycling at 40 °C and 0.1 mA·cm^−2^ (**a**) Voltage profiles; and (**b**) Evolution of the impedance spectra at 20 °C.

Cellulose is, *a priori*, not chemically inert in contact with Li metal, given the large number of protons present on the polymer backbone. Nevertheless, as in any case the stability and self repair of the SEI is the key of stable Li cycling, the use of cellulose as electrolyte separator was tested. Most cells developed dendrites, probably because of a too high and inhomogeneous reactivity. Nevertheless, in some cases, it was possible to strip and plate lithium rather steadily. In Figure 15a is represented an example of cycling behavior (although highly variable) obtained with the PYR_14_TFSI-based electrolyte. Notably, the first 10 cycles were rather stable, with no marked changes in the voltage profile and the fast obtaining of steady-state voltage. Unfortunately, after the first stop for impedance measurement, the cells became less stable with cycling and the voltage started increasing. A more stable cycling was then reached, with a moderate voltage growth with cycling. Accordingly, the impedance spectra in Figure 14b reveal a first increase of the SEI resistance, followed by the stabilization of the SEI and, in fine, a rather strong increase. One can also notice in the 100th cycle that the voltage is decreasing, which is an indication for inhomogeneous deposition.

## 3. Experimental

### 3.1. Preparation of Ionic Liquid Based Electrolytes

**Figure 16:**
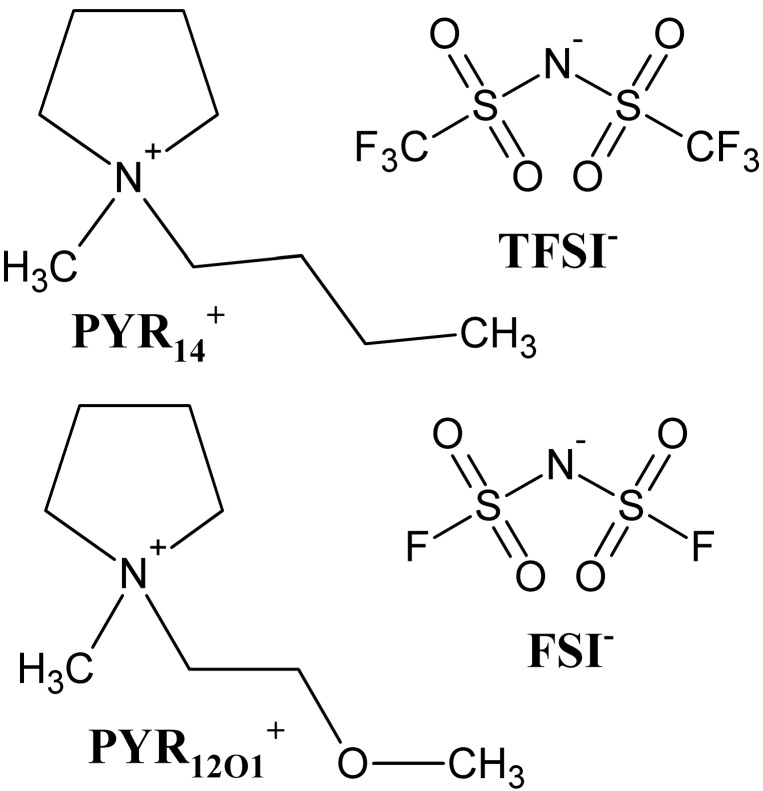
Molecular formulae of PYR_14_^+^, PYR_12O1_^+^, FSI^−^ and TFSI^−^.

Four ionic liquids (ILs) made of combinations of the *N*-butyl-*N*-methylpyrrolidinium (PYR_14_^+^) and *N*-methoxyethyl-*N*-methylpyrrolidinium (PYR_12O1_^+^), paired with bis(fluorosulfonyl)imide (FSI^−^) and bis(trifluoromethanesulfonyl)imide (TFSI^−^), shown in Figure 16. The ILs were synthesized as previously reported [46,47], using *N*-methylpyrrolidine (97%), 1-bromobutane (99%) and ethylacetate (ACS grade, >99.5 wt %) as received. 2-bromoethyl methyl ether (>85 wt %) was distilled shortly before use. All these chemicals were from Sigma-Aldrich, St. Louis, MO, USA. Potassium bis(fluorosulfonyl)imide (KFSI, Dai-Ichi Kogyo Seiyaku Co., Kyoto, Japan) and LiTFSI (3M, St. Paul, MN, USA) were used as received for IL syntheses. The resulting ILs were all colorless liquids at room temperature. Their purity was checked by ^1^H NMR for organics while ICP-OES showed halide content below 20 ppm. The ILs including PYR_12O1_^+^ (resp. PYR_14_^+^) were dried at 80 °C (resp. 100 °C) at 10^−3^ mBar for 24 h and then at *p* < 10^−7^ mBar for 24 h. The electrolytes were prepared by stirring (under vacuum < 10^−7^ mBar) at 60 °C) the pre-dried ILs and LiTFSI (dried for 24 h at *p* < 10^−7^ mBar at 120 °C) in a 9:1 molar ratio. The IL-based electrolytes were then stored in glass vessels in a desiccator inside a dry room with relative humidity below 0.1% at 21 °C.

The ionic conductivities of the IL based electrolytes, measured using a MMates AC conductimeter are listed in Table 3.

**Table 3:** Conductivity of the electrolytes at 20 °C.

Electrolyte	σ (20 °C)/S·cm^−1^
PYR_14_FSI:LiTFSI (9:1)	2.23 × 10^−3^
PYR_14_TFSI:LiTFSI (9:1)	1.07 × 10^−3^
PYR_12O1_FSI:LiTFSI (9:1)	3.94 × 10^−3^
PYR_12O1_TFSI:LiTFSI (9:1)	2.09 × 10^−3^

### 3.2. Separators Characterizations

The separators were dried under vacuum for at least 48 h, at 70 °C excepted for the glass fiber separators and Separion^®^, which were dried at 100 °C.

The separators thicknesses, listed in Table 1, were measured using a Mitutoyo micrometer thickness gauge (average of 5 measurements) while the Gurley numbers were measured using a 4110N GENUINE GURLEY Densometer (Gurley Precision Instruments, Troy, NY, USA). The measurements were repeated ten times.

Except for the air permeability measurements and SEM imaging, all measurements and cell preparations were performed inside a dry room with humidity below 0.1% at 21 °C.

The wettability of the separators was assessed by following the contact angles of electrolyte drop deposited on the surface of the separators using a Drop Shape Analysis system (DSA100S, Krüss, Hamburg, Germany). The measurements were repeated three times.

### 3.3. Electrochemical Measurements

The ionic conductivity (McMullin numbers) was measured as follow: The Celgard^®^ membranes were soaked in the electrolyte for 12 h while the other separators required only 15 min wetting prior to the conductivity measurements. Stacks from 1 to 5 separators were then placed between two stainless-steel electrodes (12 mm diameter) and their resistance was measured by electrochemical impedance spectroscopy (EIS) using a Solartron 1260 Frequency Response Analyser (Solartron Analytical, Farnborough, UK). The McMullin numbers (ratio of the resistivity of the separator soaked with electrolyte to the resistivity of the electrolyte itself, *N*m) were then calculated [48].

Symmetrical Li/(electrolyte + separator)/Li pouch cells were assembled using two 50 µm Li foils (99.999%, Rockwood Lithium GmbH, Frankfurt am Main, Germany), nickel current collectors and sealed under vacuum in pouch bag cells in the dry-room. The cells were then stored for three days at 21 °C and their impedance were monitored by EIS.

Galvanostatic Li plating/stripping tests were performed at 40 °C in a climatic chamber (MK53, Binder GmbH, Tuttlingen, Germany). A current density of 0.1 mA·cm^−2^ was applied using a S4000 battery cycler (Maccor Inc., Tulsa, OK, USA), reversing the polarity every hour. Electrochemical impedance spectroscopy (EIS) measurements were then acquired at 21 °C, after the 10th, 20th, 30th, 40th, 60th, 80th, and 100th cycles. The cycling of the cells was resumed after temperature equilibration for at least one hour after each impedance measurement.

### 3.4. Scanning Electron Microscopy (SEM) Imaging

SEM images of the separators were taken using a SEM Auriga (Carl Zeiss, Jena, Germany) after sputtering with gold. The images of the Li electrode after cycling were taken after disassembling the cells in the dry room and rinsing of the Li electrode using dimethyl carbonate. The electrodes were then transferred to the SEM chamber using a sealed cell.

## 4. Conclusions

The systematic study of separators for IL-based electrolyte ensembles allowed finding alternatives to GF separators for safe Li-ion batteries. It was shown that Celgard 2500 is a valid practical alternative to glass fiber separator for TFSI-based ILs while FSI-based IL only poorly wet polyolefin separators. Separion^®^ (or other hydrophilic separators) is thus more versatile choice for Li-ion batteries than non-functionalized polyolefin for all tested hydrophobic ILs, considering that the SEI building on graphite would not “*a priori*” be much influenced much by the separator morphology, given the limited surface area in contact and the lack of pre-existing SEI prior to the first charge. Moreover, studying the (separator + electrolyte) properties toward Li metal and Li metal cycling allowed drawing the following conclusions:

A scale for SEI-forming properties, based on the 0.1 LiTFSI-0.9 IL electrolytes behaviors with different separators could be proposed and the electrolyte with the highest conductivity, PYR_12O1_FSI, allowed the best performance. As a consequence, Separion^®^, which is not favorable for Li metal cycling when combined with TFSI-based electrolytes, due to its rough ceramic surface inducing extended damages within the “native” SEI, led to the best Li cycling results when combined with PYR_12O1_FSI, probably thanks to the formation of an effective SEI as a combination of initial SEI damage and evolution in contact with the electrolyte.

The morphology of the separator is of particular importance: Given the presence of a pre-existing SEI, the roughness of the separator influence the evolution the SEI after the initial contact, which then evolves, depending on the reactivity of the (electrolyte + separator) combination. SEM images confirmed the electrochemical results and evidenced that, in PYR_14_TFSI, a thicker SEI is deposited close to where significant damage of the SEI occurs while, in less damaged areas, the original surface morphology is barely modified.
